# Determinants of Tuberculosis treatment default in Morocco: Results from a National Cohort Study

**DOI:** 10.11604/pamj.2013.14.121.2335

**Published:** 2013-03-28

**Authors:** Nabil Tachfouti, Katia Slama, Mohamed Berraho, Samira Elfakir, Mohammed Chakib Benjelloun, Karima El Rhazi, Chakib Nejjari

**Affiliations:** 1 Laboratory of Epidemiology, Clinical Research and Community Health, Faculty of Medicine University Sidi Mohammed Ben Abdallah - Fez Morocco; 2 University Teaching Hospital – Fez Morocco; 3 Pasteur Institute of French Guiana, Epidemiology Unit

**Keywords:** Tuberculosis, Morocco, Treatment default

## Abstract

**Introduction:**

Studies have shown an association between smoking and tuberculosis (TB) infection, disease and TB-related mortality. We thus documented the impact of smoking and others factors on TB treatment default.

**Methods:**

A cohort of 1039 new TB cases matched on smoking status was followed between 2004 and 2009 in eight Moroccan regions. Treatment default was defined according to international criteria. Univariate analyses were used to assess associations of treatment default with smoking status and demographic characteristics. Multivariate logistic regression was used to adjust for potential confounding.

**Results:**

Patients’ mean age was 35.0 ±13.2 years. The rate of treatment default was 30.2%. Default was significantly higher among men, smokers, persons living in urban areas and non-religious Muslims. After adjusting for confounding variables, factors that remained significantly associated with treatment default were: being male (OR = 3.2; 95% CI: 1.2-8.7), being a non-religious Muslim (OR = 2.0; 95% CI: 1.4-2.9) and living in an urban area OR = 3.0; 95% CI: 1.8-4.9).

**Conclusion:**

The high rate found for default suggests important program's inadequacies and an urgent need for change. Therefore continued research of predictors of default and strategies to reinforce adherence is recommended.

## Introduction

Tuberculosis (TB) is a major public health problem in the world with an estimated global incidence rate of 137 cases per 100 000 population in 2009 [1]. The latest estimates are that there were almost 9 million new cases in 2011 and 1.4 million TB deaths. Geographically, the burden of TB is highest in Asia and Africa; the South-East Asia and Western Pacific Regions of which they are a part account for 60%. The African Region has approximately one quarter of the world's cases, and the highest rates of cases and deaths relative to population [2].

Providing chemotherapy to all patients diagnosed with tuberculosis is the most effective strategy for preventing the spread of tuberculosis. A World Health Assembly resolution invigorated the TB control efforts in 1991 and the internationally recommended control therapy, later named DOTS (Directly Observed Therapy Short-course), was launched in 1994 [3].

A major barrier to better results is the high number of new smear-positive cases that voluntarily interrupt treatment (treatment defaulters). Adherence to the long course of TB treatment is a complex phenomenon with a wide range of factors impacting on treatment taking behavior. Treatment default is detrimental for both patient and community, through higher risks of relapse [4], lower therapeutic successes in case of retreatment [5, 6], increased risk of developing multi-resistant strains and higher risk of death [7–10].

In resource-constrained settings where the health care services are not well developed, delayed presentation for treatment and defaulting from treatment are the two major challenges that TB programs face [11]. A recent retrospective study reported a default rate of 20% in Cameroun; the same incidence was reported in smear-positive pulmonary TB patients in Southern Ethiopia [12–13].

In Morocco, TB is highly prevalent: it affected almost 27000 people in 2010, with an incidence of approximately 82 new cases per 100000 populations (Health Ministry Statistics) National TB treatment guidelines in 2007 and 2008 recommended a Category I treatment regimen- 2 months of isoniazid, rifampin, pyrazinamide, and streptomycin followed by 4 months of rifampin and isoniazid (2SHRZ/4RH)- for new smear-positive cases and a Category II regimen- 2HRZES/1RHEZ/5RHE (E = ethambutol)- for retreatment cases. Beginning in 2009, ethambutol replaced streptomycin in Category I regimens [14]. TB cases are initially managed in specialized centers for tuberculosis diagnosis, dependent on the Ministry Of Health, and then referred to Tuberculosis Control Units (TCU) for the treatment. Treatment is free of charge, and dispensed under medical surveillance in 2600 urban and rural health centers, following the DOT strategy. As a result of the efforts of the Moroccan government to fight TB, numbers are slowly decreasing by 2 to 3% annually (Health Ministry Statistics).

Despite the absence of fees to pay for treatment, a high number of new smear positive cases voluntarily interrupt their treatment before the end. Treatment default is a major obstacle in the fight against TB, and was estimated around 10% in 2009 [15]. There is little data on the determinants of TB treatment default in the specific context of Morocco. Ensuring successful treatment completion might require addressing multiple factors beyond simple supervision of drug intake [16–17]. Better knowledge of the profile of treatment defaulters may help control programs to identify those likely to benefit from targeted interventions and can be helpful in improving compliance.

Tobacco smoking is a risk for TB infection, disease and mortality. Smoking appears to be related to other factors such as delay for treatment and smear conversion during treatment (Slama et all 2007). The evidence for a connection between smoking and default, however, is inconclusive. Based on the premise that smokers tend to be less compliant to medication [18, 19], we hypothesized that smokers with TB disease are less likely to comply with their TB treatment. We aim to document the impact of smoking and others socio demographic factors on adherence to treatment among Moroccan TB patients.

## Methods

**Setting:** The study was conducted in 15 Tuberculosis Control Units (TCUs) in public primary and respiratory care centers in urban settings; TCUs were selected based upon their case load, accessibility and physicians’ willingness to participate. Participating TCUs were located in eight administrative regions representing more than half of the total population and reflecting the ethnic and sociodemographic characteristics of the entire Moroccan TB patient population. The 15 TCUs cover more than 60% of the eight regions’ general population.

**Design:** A cohort of new cases of TB diagnosed between January 2004 and December 2008 was assessed at the beginning of treatment and at five intervals between inclusions until the end of follow-up.

**Study population:** Eligible patients included all previously untreated patients (new cases) who were enrolled in treatment for tuberculosis (TB) during the designated period of recruitment. We recruited male or female patients aged between 18 and 79 years, who had evidence of active tuberculosis, defined according to World Health Organization standards and accepted to take part into study [20]. Pulmonary TB (PTB) was diagnosed in patients having symptoms of fever or cough and a sputum smear that showed acid-fast bacilli or a chest radiograph that showed changes compatible with a diagnosis of tuberculosis. We excluded patients who had other severe underlying disease or were receiving concomitant corticosteroid or immunosuppressive therapy. A written informed consent to participate in the study was obtained before administration of the questionnaire, and the study protocol and patient information sheet were approved by the Ethics Committee of the Moroccan Health Ministry.

**Study conduct and data collection:** All patients received combination antituberculous drug treatment and ancillary clinical care and follow-up for tuberculosis according to National program recommendations and international guidelines [12]. A face to face questionnaire was developed in collaboration with the International Union against Tuberculosis and Lung Disease (The Union). A French version of the questionnaire was then developed by the Department of Epidemiology and Public Health in Fez with the collaboration of The Union. The questionnaire was also translated into Moroccan Arabic dialect. Slight modifications in the wording were made to correct and clarify some discrepancies in meaning. The questionnaire focused on the following topics: 1) socio demographic characteristics (age, sex, family income, and educational level), 2) smoking status: smokers were those who had smoked at least 100 cigarettes in their lifetime and who still smoked daily or occasionally (current smokers) at the time of TB diagnosis or had recently stopped (for less than 3 months). Non-smokers were those who had never smoked or had smoked fewer than 100 cigarettes in their lifetime, but had quit for more than three months. The questionnaire was administered by chest specialists (pneumologists) and general practitioners who were given a 2-day training course on background and theoretical questions and in the correct use of the protocol and questionnaires. All of the treatment outcomes of patients who started treatment during the recruitment phase of the study were ascertained by TCU physicians. Outcomes were defined by international consensus: defaulters were defined as patients being off drugs for more than eight weeks before the end of the treatment period, and included also all patients lost to follow-up in the first 4 months of the study period [21, 22].

**Data management and statistical analysis:** Descriptive statistics were used for patients' demographic information. Student's t-test and Chi-squared test were applied to determine demographic and smoking status differences in treatment default among the respondents; significance was attributed to a probability of p<0.05. Multivariate logistic regression analysis was used to adjust for potential confounding factors. Variables associated with the outcome with a p value <0.05 were included in a step-wise multiple logistic regression analysis. Crude and adjusted ORs and 95% CIs were calculated.

## Results

A total of 1039 TB patients (536 smokers and 503 non-smokers) were identified for potential selection. No data was available among persons who refused to take part in the survey. The patients’ mean age was 35.0 ± 13.2 years; 45.9% were aged <‘180) for 71% of the population. More than half (51.6%) of participants were smokers due to the recruitment strategy for the original trial. Seventeen percent of patients had an extra pulmonary form of tuberculosis. Table 1 shows repartition of study population according to sociodemographic characteristics.

**Table 1:** Sociodemographic and economic characteristics of the study population

Variable	Percentage (%)
**Age (N= 1012)**	
<30	45.9
≥30	54.1
**Gender (N= 1027)**	
Men	95.7
Women	4.3
**Educational level (N=975)**	
Illiterate	22.5
Basic/primary	30.5
Secondary	21.0
Undergraduate degree	23.4
Other	0.6
**Residential area (N=1016)**	
Urban	77.1
Rural	22.9
**Household monthly income (N=888)**	
<1000	38.4
1000–2000	33.0
2000–4000	13.2
4000–6000	12.7
>6000	2.7
**Religion (N=612)**	
Religious Muslim	49.7
Non-Religious Muslim	50.3
**Clinical form of TB (N=986)**	
Pulmonary	82.6
Extra pulmonary	17.4

Treatment default rate was 30.2%. One third of the defaulters (32.2%) interrupted treatment during the first two first months (intensive phase), 22% during the second phase, and almost 46% during the final 2 months of treatment as shown in Table 2.

**Table 2:** Time of default from treatment among the 81 defaulters

Time of default from treatment	Frequency number	Cumulative frequency number
2^nd^ month	101 (32.2)	101 (32.2)
4^th^ month	69 (22.1)	170 (52.2)
6^th^ month	143 (45.7)	313 (100)

In the univariate analysis, factors associated with TB treatment default included being male (odds ratio OR = 2.8; 95%; CI: 1.2-6.6), living in an urban area (OR = 2.8; 95%; CI: 1.9-4.1). Tobacco smoking (OR = 1.3; 95% CI: 1.1-1.5), having an education of more than 6 years (OR = 1.3; 95% CI: 1.1-1.9) and being a non-religious Muslim (OR = 2.01; 95% CI: 1.4-2.9). Characteristics such as age, monthly income and clinical form of TB were not associated with TB treatment default. Table 3 shows patient characteristics associated with TB treatment adherence.

**Table 3:** Patient characteristics associated with TB treatment adherence

Characteristics	Defaults	Non-default	OR (95% IC)	P
**Gender (N=1027)**				
Men	98.0	94.7	2.8 (1.2–6.6)	0.01
Women	2.0	5.3		
**Age (N=1035)**				
<30	48.2	44.9	1.1 (0.8–1.4)	NS
≥30	51.8	55.1		
**Smoking status (N=1039)**				
Smokers	56.1	49.7	1.3 (1.1–1.5)	0.05
Non-smokers	43.9	50.3		
**Education (N=996)**				
< 6years	47.9	57.7	1.3 (1.1–1.5)	<0.01
≥6years	52.1	42.3		
**Residence area (N=1016)**				
Rural	12.0	27.5	2.8 (1.9–4.1)	<0.001
Urban	88.0	72.5		
**Religion (N= 612)**				
Religious Muslim	62.4	45.1	2.01 (1.4–2.9)	<0.001
Non-religious Muslim	37.6	54.9		
**Clinical form of TB (N=986)**				
Pulmonary	85.0	81.5	0.8 (0.5–1.1)	NS
Extra pulmonary	15.0	18.5		

In the multiple logistic regression analysis, only being male (OR = 3.2; 95% CI: 1.2-8.7), being a non-religious Muslim (OR = 2.0; 95% CI: 1.4-2.9) and living in urban area OR = 3.0; 95% CI: 1.8-4.9) were independently associated with non-adherence when adjusted for age and smoking status. Table 4 shows multivariate logistic regression analysis of the association between TB treatment adherence and sociodemographic characteristics among the study participants

**Table 4:** Logistic regression analysis of the association between treatment non-adherence and sociodemographic characteristics among the study participants

Characteristics	Adjusted OR (95% IC)	p
**Gender**		
Men	3.2 (1.2–8.7)	0.02
Women	Referent	
**Residence area**		
Urban	3.0 (1.8–4.9)	<0.001
Rural	Referent	
**Age**		
<30	1.0 (0.6–1.4)	NS
≥30	Referent	
**Smoking status**		
Smokers	0.7 (0.5–1.1)	NS
Non-smokers	Referent	
**Religion**		
Religious Muslim	2.0 (1.4–2.9)	<0.001
Non-religious Muslim	Referent	

## Discussion

This study is the first in Morocco to measure the incidence of antituberculosis treatment discontinuation and predictors of treatment discontinuation in a large cohort of patients treated for tuberculosis.

The default rate of 30% in your study was similar than the 29% reported from Zambia [23] and Nigeria (24). However, it was higher than the 20% default rate reported in South Ethiopia and Cameroon [12, 13].

Our study counted all patients as defaulters if they were not seen at all follow-up periods. It should be noted that almost half of the default rate is assessed at the end of the continuation phase, which normally would be defined as loss to follow-up. Nevertheless, treatment non completion is required to fall below 10% in order to achieve treatment success of 85%, one of the health-related indicators of the Millennium Development Goals [25], and our results indicate an important problem that needs urgently to be addressed.

Concerning temporal trend of the default rate, about 68% of the patients defaulted during the continuation phase of treatment. This finding was higher than the rate observed from Nigeria (23%) [24], it was similar to those seen in Cameroon (62%) [13] and Kenya (57%) [26], but lower than the findings from Ethiopia where 91% of the patients defaulted during the continuation phase [12]. One possible explanation for the early default could be feeling better shortly after initiation of treatment. Inadequately counseled patients may mistake the feeling of improvement to cure, thus stop medication early. Feeling better was cited among reasons for default and has similarly been reported in other studies as cause for default [17, 27]. Adequate patient education and counseling at initiation of treatment is therefore important and could be implemented at the national program.

The high default rate may be related to the recruitment procedure of matching non-smokers and smokers. We do not believe this to be the case, because the sample was similar, apart from the proportions of smokers, to other data found for the general population of Moroccan TB patients seen in treatment [6].

The multiple logistic regression analysis showed that, after adjusting for confounding factors, gender is the strongest predictor of non-compliance, followed by residence in an urban area and the absence of religious practice. Male gender has often been found to be a risk factor for non-compliance to tuberculosis treatment. In Turkey, a retrospective review has found a higher rate of default among males than (41.6% versus 31.8% among women) [28]. In a Syrian Arab Republic study, after adjustment for confounders, male sex was found to be a significant risk factor for a negative treatment outcome (default, failure or death) with a 2.9-fold increased risk [29]. The same finding was reported in recent studies in Nigeria and Kenya [24, 26]. Specifically in Morocco, a recent study showed a higher risk of failure, default, or relapse within two years for male gender (OR = 2.29, 95% CI 1.10-4.77) [6]. Males may be less compliant to TB treatment for economic reasons. In various cultures, men are the main contributors to the family income and cannot afford to take time out for a medical visit to a clinic. Indeed, travel time to and from the clinic represents time absent from work and potentially less money earned [30].

Non-compliance to TB treatment was also associated to living in an urban area. It has been suggested that patients living in urban settings may be less compliant to TB because, in busy urban clinics, time for education may be limited [31, 32]. An Estonian study of risk factors for treatment default in pulmonary tuberculosis also found urban residence to be an independent risk factor for treatment default (OR = 1.85, 95%CI 1.00-3.42) [33]. It is possible also that the numerous attractions in urban environments might be a distraction from regularly following the tuberculosis treatment. Conversely, in Nigeria, rural residence was a predictor of treatment default in tertiary hospital, certainly due to distance from home to urban clinics [24].

Religious Muslims appeared to be more adherent to treatment than non-religious Muslims. Non-religious Muslims have also been found to be less likely to adhere to healthy behaviors such as smoking cessation. Religious faith and compliance to HIV treatments have also been found. A study of HIV-infected youth aged 14-22 showed that subjects who had excellent adherence had significantly higher religious belief scores than those who had poor adherence [34]. A study of HIV patients in Uganda showed a significant relation between anti-retroviral therapy adherence and religiosity [35].

A number of limitations in this survey have to be recognized. We may have introduced bias by combining patients who stopped smoking upon discerning symptoms of TB, with those who had never smoked. Self-reporting of smoking status could have been biased by a certain degree of dissimulation of smoking behaviour because of the social desirability for patients not to be smokers. In Morocco, addictive behaviors such as tobacco, alcohol or hashish use may have been underreported because data were collected using face to face questionnaires. Patients may also have been uncomfortable reporting their religious practices. Third, in this cohort, women represented only 4.4% of the TB cases. Because the cohort was designed to evaluate the impact of smoking on treatment outcomes, the study design required that half of the participants be smokers, limiting the number of females in the study. This imbalance was also influenced by smoking rates in Morocco, where smoking is very uncommon among women [36].

Finally, we did not assess the impact of other factors which were reported as predictors of treatment default in other area. Mainly human immunodeficiency virus (HIV) status, herbal medication use, knowledge on tuberculosis and distance from home to clinic [24, 26, 37, 38].

On the other hand, our convenience sample of primary health care units was representative of the overall TCU attendance with respect to basic socio-demographic factors and geographic repartition in the Moroccan territory.

## Conclusion

This analysis documents some of the personal and social risk factors for treatment non-compliance in Morocco. The factors that we found associated with default have been found elsewhere: male sex, urban residence, absence of religious practice. Smoking was not found to be related to lower adherence to treatment. The high rate found for default suggests important program's inadequacies and an urgent need for change. Therefore continued research of and strategies to reinforce adherence is recommended. Especially, other predictors should be evaluated in subsequent studies.
